# Development of BacMam Induced Hepatitis E Virus Replication Model in Hepatoma Cells to Study the Polyprotein Processing

**DOI:** 10.3389/fmicb.2020.01347

**Published:** 2020-06-18

**Authors:** Manjeet Kumar, Preeti Hooda, Madhu Khanna, Utkarsh Patel, Deepak Sehgal

**Affiliations:** ^1^Virology Laboratory, Department of Life Sciences, Shiv Nadar University, Greater Noida, India; ^2^Virology Lab, Department of Virology, Vallabhbhai Patel Chest Institute, University of Delhi, New Delhi, India

**Keywords:** HEV, BacMam, *in vitro* culture, polyprotein, processing, replication

## Abstract

The processing of polyprotein(s) to form structural and non-structural components remains an enigma due to the non-existence of an efficient and robust Hepatitis E Virus (HEV) culture system. We used the BacMam approach to construct an HEV replication model in which the HEV genome was cloned in the BacMam vector under the CMV promoter. The recombinant BacMam was used to infect Huh7 cells to transfer the HEV genome. HEV replication was authenticated by the presence of RNAs of both the polarity (+) and (−) and formation of hybrid RNA, a replication intermediate. The presence of genes for Papain-like Cysteine Protease (PCP), methyltransferase (MeT), RNA dependent RNA polymerase (RdRp), and ORF2 was confirmed by PCR amplification. Further, the infectious nature of the culture system was established as evidenced by the cross-infection of uninfected cells using the cell lysate from the infected cells. The HEV replication model was validated by detection of the ORF1 (Open Reading Frame1) encoded proteins, identified by Western blotting and Immunofluorescence by using epitope-specific antibodies against each protein. Consequently, discrete bands of 18, 35, 37, and 56 kDa corresponding to PCP, MeT, RdRp, and ORF2, respectively, were seen. Besides demonstrating the presence of non-structural enzymes of HEV along with ORF2, activity of a key enzyme, HEV-methyltransferase has also been observed. A 20% decrease in the replicative forms of RNA could be seen in presence of 100 μM Ribavirin after 48 h of treatment. The inhibition gradually increased from 0 to 24 to 48 h post-treatment. Summarily, infectious HEV culture system has been established, which could demonstrate the presence of HEV replicative RNA forms, the structural and non-structural proteins and the methyltransferase in its active form. The system may also be used to study the mechanism of action of Ribavirin in inhibiting HEV replication and develop a therapy.

## Introduction

Hepatitis E virus (HEV) is an emerging virus, transmitted via the fecal-oral route through contaminated drinking water (Abravanel et al., 2015). Due to poor sanitation, it is more prevalent in developing countries (Cao and Meng, 2012), though HEV cases in developed countries are also on the rise (Minuk et al., 2007; Dalton et al., 2008; Mushahwar, 2008). HEV has a mortality rate of 3% affecting 20 million people annually (Jameel, 1999), while it increases up to 30% in the third trimester of pregnancy due to liver failure (Navaneethan et al., 2008; Aggarwal and Naik, 2009). HEV is a small, non-enveloped virus having single-stranded RNA of positive-sense which is ∼7.2 kb in length and has three open reading frames; ORF1, ORF2, and ORF3 (Tam et al., 1991; Tsarev et al., 1992; Ahmad et al., 2011). An ORF4 has also been seen in genotype 1 strain of virus (Nair et al., 2016). ORF1 being the largest open reading frame codes for a non-structural polyprotein of ∼186 kDa, which is required for viral survival and its replication (Ansari et al., 2000). Using computational homology analysis by Koonin et al. (1992), the polyprotein has been predicted to have the domains that code for the MeT, Hel, PCP, and RdRp. The study of the processing of these enzymes from the polyprotein (ORF1) has been the focus of the present study (Koonin et al., 1992). Besides, the viral genome includes the Y domain (Y) (Paliwal et al., 2014; Parvez and Khan, 2014; Parvez, 2017), a proline-rich hypervariable region (H), and the X -domain (X). The second ORF, ORF2 encodes for the Viral Capsid protein, while HEV ORF3 translates to a phosphoprotein that may be responsible for infection and the viral egress (Graff et al., 2005; Chandra et al., 2008; Yamada et al., 2009a).

A block in the study of the HEV is the lack of availability of the effective *in vitro* culture system, and this has posed a challenge in understanding its replication, processing or drug therapy (Kenney and Meng, 2019; Todt et al., 2020). Many attempts have been made to create an efficacious culture system in the past. In one of the studies, 21 hepatic and non-hepatic cell lines were transfected with a viral strain to conclude PLC/PRF/5 as the most viable and responsive cell line (Tanaka et al., 2007). In another study, a high virus load of 2.0 × 10^7^ copies/ml was achieved when the cells were infected with the virus from a Japanese patient with acute hepatitis E (strain JE03-1760F) GT3 (Tanaka et al., 2007; Okamoto, 2011). It has been observed that the efficiency of the cell culture system rests on the type of cell line, a strain of the virus, and the medium used for the growth of the virus (Schemmerer et al., 2019). Other viral strains attempted for enhanced viral propagation include GT4 HE-JF5/15F, JE03-1760F, Sar-55/S17 or Kernow-C1/p6 could achieve a high viral load up to 2.0 × 10^7^ copies/ml (Emerson et al., 2004; Tanaka et al., 2007; Takahashi et al., 2012; Shiota et al., 2013). In a recent study, different regions of HEV, 14-16753 (3c), 14-22707 (3e), and 15-22016 (3f-like) were used to achieve a significantly high viral titre with 10^8^, 10^9^, and 10^6^.^5^ HEV RNA copies/ml (Schemmerer et al., 2019). In another strategy, the strain of HEV genotype 3 p6 (Kernow C-1) was used to infect the human liver cell lines HepG2 and HepG2/C3A which were grown in different media to produce a high titre with 10^5^ and 10^6^ FFU/ml of the virus (Todt et al., 2020). However, many culture systems having high titre have been reported in recent past, not much progress has been made to understand the proteins or the enzymes and their role in replication. This besides that Koonin predicted putative domains of HEV in 1992. However, functional studies on HEV proteins and its enzymes remains a pre-requisite to understand the phenomenon of replication, translation, ingress, and the egress.

The present study is an attempt to demonstrate the processing of the polyprotein into smaller fragments of structural and non-structural proteins. We detected three of the four enzymes and demonstrated the activity for one of these. Few remote studies conducted so far remain inconclusive and unsubstantiated for understanding the existence and role of the viral enzymes. In past, a contradictory study concluded that the ORF1 could release the proteolytic fragments of 107 and 78 kDa in HepG2 cells (Ropp et al., 2000), but the claim was retrieved 10 years later (Suppiah et al., 2011). In some other studies, polyprotein processing has also been determined partially or wholly (Magden et al., 2001; Paliwal et al., 2014; Parvez and Khan, 2014) but the observations are yet to be confirmed. Other heterologous expression systems that include *Escherichia coli* (Behloul et al., 2017), Yeast (Ojha and Lole, 2016), and cell-free (Nan and Zhang, 2016) have been tried to express the viral proteins, but no significant cleavage or the enzyme activity could be seen. In another study, ORF1 got processed into three fragments of sizes 38, 36, and 35 kDa, which were detected through polyclonal antibodies against MeT, Hel, and RdRp, respectively (Panda et al., 2000) but the results need to be validated using monoclonal or the domain-specific antibodies. However, Sehgal et al. (2006) showed the ORF1 processing into eight fragments which got arrested by inhibitor, E-64d, validating the processing of polyprotein by the Protease. In our present study, we have found the fragments of size 18, 35, 37, and 56 kDa, which according to computational modeling (Koonin et al., 1992) and antibody staining represent MeT, PCP, RdRp, and ORF2, respectively.

In the present study, we could identify the translated products of the polyprotein. Further, the *in vitro* culture system has been validated for its transcriptional and translational property since the inhibition of the viral replication in cross-infected cells was found to decrease by ∼20% when treated with 100 μM of Ribavirin. However, the effect of the Ribavirin-induced mutagenesis of the hepatitis E virus genome has been earlier shown (Todt et al., 2016). Another drug, Sofosbuvir has been seen to be effective against the HEV genotype 1 replicon by the reduction of the replicon RNA levels (Netzler et al., 2019). In the present study, we used the BacMam approach to transfer the HEV genome into the Huh7 cells since it has been used earlier for the expression of heterologous proteins under the CMV promoter in the mammalian cells (Kost et al., 2005; Koroleva et al., 2010). Besides, BacMam-mediated gene transfer into various cell lines of hepatic origin has also been successful (Hofmann et al., 1995; Boyce and Bucher, 1996; Shoji et al., 1997; Hüser and Hofmann, 2003; Chen et al., 2005). Using this strategy, the Huh7 cells were infected with a recombinant Baculovirus integrated with HEV genome under CMV promoter. This process circumvented the need for harsh treatments like chemical mediated transfection or electroporation. Upon entry of the recombinant BacMam-HEV into Huh7 cells, the viral genome was expressed to form the new viral particles by identifying the (+) and (−) stranded RNA by amplification of a region of the capsid protein, ORF2. A significant implication of any culture system has been the testing of drugs and the inhibitors that can stop or decrease viral replication. The only off-shelf drug that has been tested in antiviral therapy happens to be Ribavirin (Todt et al., 2018). We tested Ribavirin on the cross infected culture at 24 and 48 h to find the decrease in RNA copies as a function of time. An exhaustive study is required to establish a more efficient culture system further, to study the enzymes and establish the role of drugs using the system.

## Results

### Construction of Recombinant BacMam Carrying HEV Genome

**FIGURE 1:**
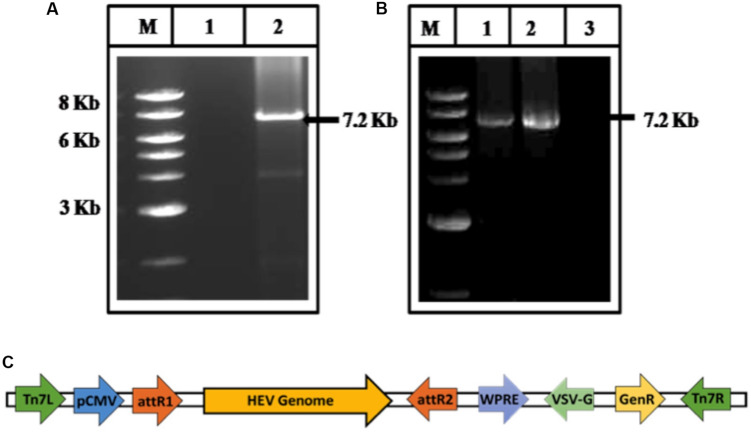
Cloning of HEV genome **(A)** amplification of HEV genome from cDNA of pSHEV-3. The corresponding lanes are as follows: marker (lane M), No template control (lane 1), amplified pSHEV-3 product (Lane 2). **(B)** PCR amplification of HEV genome from BacMam-HEV construct. The corresponding lanes are as follows: Marker (lane M), Positive Control (amplified cDNA of pSHEV-3) (lane 1), an amplified product from BacMam-HEV construct (lane 2), No template control (lane 3). **(C)** Schematic representation of the BacMam-pCMV-Dest vector carrying the HEV Genome.

As a first step in constructing recombinant BacMam carrying HEV genome, the HEV cDNA was amplified from the plasmid, pSHEV-3, a kind gift from Meng, using gene-specific forward (GT3pDONRF1) and the reverse primer (GT3pDONRR1) (Supplementary Table S1) (Figure 1A). The amplified product was cloned in the vector, pDEST-BacMam (Life Technologies, United States) having a CMV promoter and confirmed through PCR (Figure 1B). The gene cloning cassette map is given below (Figure 1C) which includes vesicular stomatitis vascular glycoprotein (VSVG) and woodchuck post-transcriptional regulatory elements (WPRE), known to enhance RNA stability and enhance the gene expression, respectively (Barsoum et al., 1997; Kantor et al., 2014).

### Time Course Expression of Viral RNA

**FIGURE 2:**
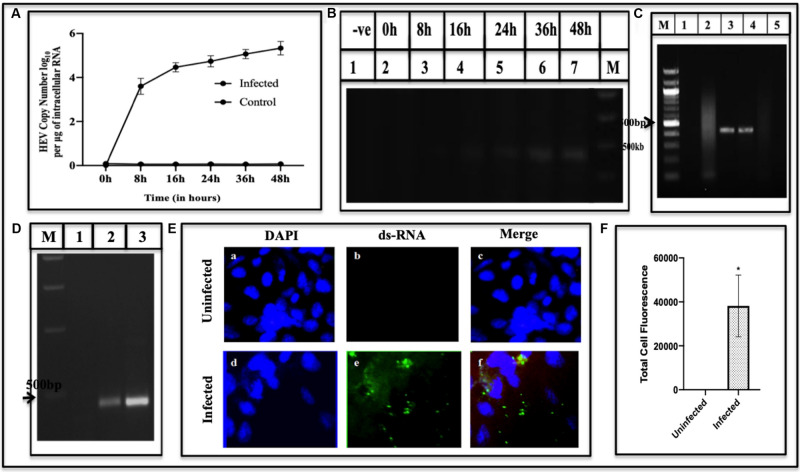
Quantification and detection of viral RNA. **(A)** Viral RNA copies in Huh7 cells were quantified at different time points (0–48 h) using qPCR. The graph indicates the HEV copy number in log_10_ per microgram of total intracellular RNA. Error bar indicates the standard deviation. **(B)** Amplification of cDNA generated by RT-PCR of viral RNA isolated from infected cells at the corresponding time using primers GT3RTF and GT3RTR (Supplementary Table S1). The amplified products were fractionated on 1.4% agarose gel **(B)**. Lane M; Marker, Lane 1, Uninfected; 2–7 indicate amplified product from infected cells at different time points (0–48 h). Detection of Negative and Positive Strand of RNA. **(C)** RNA supernatanted from the cellular lysate of Huh7 infected cells followed by DNase treatment was used as a template for PCR using primer against ORF2 region (Supplementary Table S1). The corresponding lanes are as follows: Lane M, Marker; Lane 1, uninfected cells RNA followed by cDNA synthesis used as a template; Lane 2, infected cells RNA sample with DNase treatment; Lane 3, infected cells RNA with DNase treatment followed by cDNA synthesis and used as a template; Lane 4, GT3 Plasmid as a positive control; Lane 5, no template control. **(D)** Detection of negative and positive strand of viral RNA using strand-specific reverse transcription-PCR (RT-PCR). The corresponding lanes are as follows: Lane M, Marker; Lane 1, mock; Lane 2, negative-strand; Lane 3, positive-strand. Visualization of dsRNA in infected cells using Immunofluorescence. **(E)** Cells were stained with anti-dsRNA (J2 Monoclonal Antibody) detected with Alexa Fluor 488 Rabbit Anti-Mice IgG secondary antibody and counterstained with DAPI to stain nuclei. Panels **(a–c)** represent uninfected Huh7 cells and panels **(d–f)** represent HEV infected cells. Panels **(c,f)** represent the merged image. **(F)** Quantification of fluorescence. It was done using ImageJ Software. The graph represents the mean fluorescence intensity, and the error bar indicates the standard deviation. The statistical significance of data was tested using the Mann–Whitney test (*p*-value = 0.0286). * indicates *p*-value < 0.05. All experiments were performed at least three times as independent experiments.

Time course of HEV replication in the culture system was studied by detecting the amount of RNA and determining the polarity of RNA copies transcribed in the infected cells at different time points. For this, Huh7 cells were infected with the recombinant baculovirus (MOI > 100), and the RNA from infected cells was extracted and reverse transcribed to cDNA. The copies were calculated at 8, 16, 24, 36, and 48 h post-infection using RT-PCR (Figure 2A) and fractionated on 1.4% agarose gel (Figure 2B). RNA copy number was plotted in log_10_ per μg of intracellular RNA against different time points. The RNA copy number at 8 h post-infection was found to be 8.3 × 10^3^, which increased up to 5.2 × 10^5^ at 48 h (Figure 2A) and then declined (data not shown). The study concluded that the maximum copies of HEV RNA in the cells were observed between 36–48 h. The results were obtained by amplifying the representative ORF2 gene fragment using HEV-ORF2 primers (Supplementary Table S1).

### Detection of RNA Species During HEV Replication

Three species of RNA have been identified, in the past, that appear at different stages of the viral replication viz. positive RNA, negative RNA synthesized from positive RNA, and the hybrid of negative and the positive RNA as an intermediary product. The Huh7 cells were used in the study since they are replicative competent (Emerson et al., 2004) and more efficient, as compared to other cells, to affect post-translational modifications (Dumont et al., 2016). To identify RNA replicative forms during HEV replication, Huh7 cells were infected by recombinant BacMam-HEV at MOI of ∼100. For identifying the negative and positive sense RNA, the cDNA transcribed from the infected cell lysate was amplified using forward and reverse primer of the internal gene, ORF2 (Supplementary Table S1) (Figure 2D). To ensure that the DNA of recombinant baculovirus does not get amplified, PCR was performed using DNase treated RNA as a template (Figure 2C). To analyze the intermediate replicative form of RNA, the double-stranded RNA, J2 Monoclonal antibody was used, which specifically bound to dsRNA. It was detected with Alexa Fluor 488 Rabbit Anti-Mice secondary antibody (Weber et al., 2006) (Figure 2E). The fluorescence from infected cells was quantified using ImageJ software. The statistical significance of data was tested using the Mann–Whitney *U* test (Figure 2F).

### Cross-Infectivity of *in vitro* HEV Culture

**FIGURE 3:**
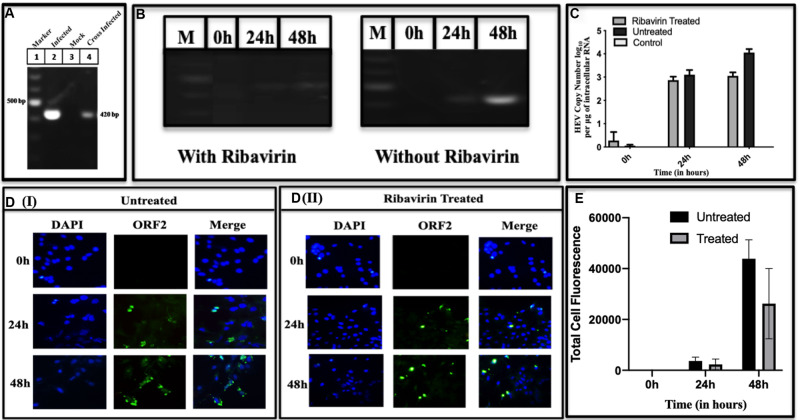
Cross-infectivity of HEV in Huh7 cells. Lysate of the cells infected with BacMam-HEV adsorbed on the uninfected Huh7 cells. **(A)** RT-PCR for HEV ORF2 region. The corresponding lanes are as follows: Marker (lane 1), BacMam-HEV infected cell lysate (lane 2), control cells lysate (without infection) (lane 3), Huh7 cells adsorbed by the cell lysate of virally infected cells (lane 4). **(B)** It represents the RT-PCR of Ribavirin treated and untreated cells at 0, 24, and 48 h, respectively. M represents Marker. **(C)** qPCR was used for quantification of HEV copy number of uninfected, Ribavirin Treated and Untreated Cells. Statistical significance of Data was tested using 2-way ANOVA. The data was statistically significant as the *p*-value < 0.05 (*p*-value 0.0019, 0.0039, <0.0001). All the experiments were done in duplicates, and the error bar indicates the standard deviation. The values represent the mean of three independent experiments. **(D)** Immunofluorescence at different time points. Panels **(DI,DII)** show the intensity of ORF2 at 0, 24, and 48 h in cross-infected cells without Ribavirin treatment and with Ribavirin treatment, respectively. The cells were stained with Anti-ORF2 primary antibody, which was detected with Alexa Fluor 488 goat Anti-Rabbit IgG secondary antibody. **(E)** Quantification of fluorescence. The intensity of fluorescence was quantified with ImageJ software. Total four cells were selected to quantify the fluorescence intensity. The bar graph represents the mean fluorescence intensity, and the error bar indicates the standard deviation. The statistical significance of data was tested by GraphPad prism using 2way ANOVA. (*p*-value = 0.0247, <0.0001, 0.0278) *p*-value < 0.05 is considered as significant. All the experiments were performed at least three times as independent experiments.

To confirm cross-infectivity of the HEV replicated in Huh7 cells, the assay was used as described in earlier studies (Parvez et al., 2011; Córdoba et al., 2012). For this, the cell lysate from infected Huh7 cells was overlaid on the uninfected cells and RNA was extracted after 48 h of infection. The cDNA amplified from the cross-infected cells was fractionated on the agarose gel. Figure 3A lane 2 represents the BacMam-HEV infected Huh7 cell lysate, while lane 3 represents cross-infected cells. A band of the size of ∼420 bp is seen in both the lanes indicated that the replicating viral RNA was present in the cross-infected cells. The cross-infectious property of the viruses produced in infected Huh7 cells was tested by studying the effect of Ribavirin on the RNA replicative forms. More copies of viral RNA were found in the cells without treatment of Ribavirin while a decrease was seen in Ribavirin treated cells (Figures 3B,C). This indicated that the Ribavirin could inhibit or decrease the HEV copy number in the cross-infected cells. In order to see the Ribavirin effect at cellular level, cross-infected Huh7 cells were treated for 24 and 48 h with Ribavirin to find the decrease in copy number in a time-dependent manner (Figure 3C). The effect of Ribavirin was further validated by using indirect Immunofluorescence at 0, 24-, and 48-h post-infection. The infected Huh7 cells (Ribavirin treated and untreated) were stained with ORF2 antibodies and detected using Alexa Flour 488 Anti-Rabbit secondary antibody. Reduction in fluorescence intensity was observed in Ribavirin treated cells [Figure 3D(II)] with respect to untreated cells [Figure 3D(I)]. The fluorescence intensity was quantified using ImageJ software and the statistical significance of data was tested using 2-way ANOVA (Figure 3E).

### Expression of Non-structural and Structural Proteins

#### Methyltransferase

**FIGURE 4:**
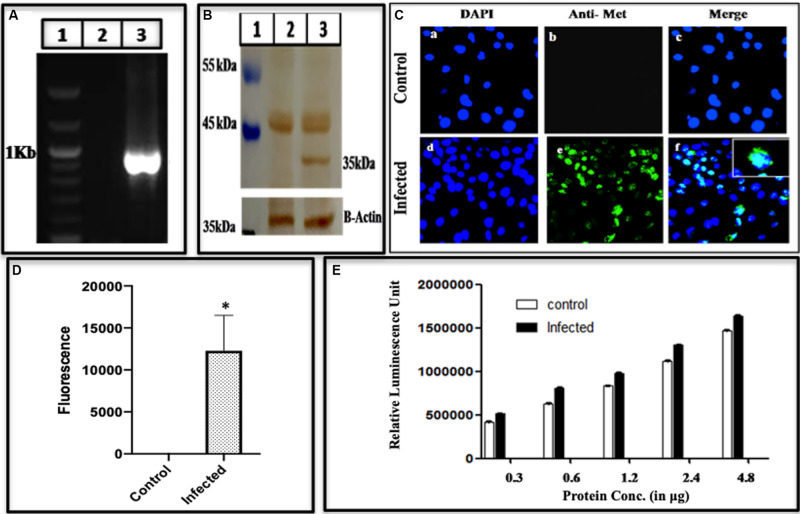
Expression of active methyltransferase. **(A)** PCR Amplification of methyltransferase from BacMam-HEV infected cells using primers G3MeTF and G3MeTR (Supplementary Table S1). The corresponding lanes are as follows: Marker (lane 1), cDNA from uninfected cells (lane 2), cDNA from infected cells (lane 3). **(B)** Western blot analysis using anti-methyltransferase antibody and Goat Anti-Rabbit IgG HRP conjugated secondary antibody. The corresponding lanes are as follows: Marker (lane 1), cell lysate from Huh 7 control cells (lane 2), cell lysate from infected Huh 7 cells showing specific band identical to the size of methyltransferase (lane 3). **(C)** Immunofluorescence assay: cells were stained with Anti-MeT detected with Alexa Fluor 488 Goat Anti-Rabbit IgG secondary antibody and counterstained with DAPI to stain nuclei. Panels **(a–c)** represent control Huh7 cells, and panels **(d–f)** represent HEV infected cells. Panels **(c,f)** represent the merged image. The Immunofluorescence was performed three times as independent experiments. **(D)** The graph represents the mean fluorescence intensity, and the error bar indicates the standard deviation. Statistical significance of data was determined by the Mann–Whitney test (*p*-value = 0.0286). * indicates *p*-value < 0.05. **(E)** Methyltransferase activity using MTase-Glo reagent. The histogram shows an increase in methyltransferase activity upon an increase in enzyme concentration. The activity was performed three times in triplicates and the bar indicates the standard deviation. The statistical significance of data was tested using 2 way ANOVA. The data was found to be significant as the *p*-value < 0.05 (*p*-value < 0.0001, <0.0001, <0.0001).

Methyltransferase is the first enzyme on the viral genome that is required for capping at 5′ end, and has been demonstrated to be present in active form in the current study. Primarily, total cellular RNA from infected cell lysate was prepared, converted to its cDNA and amplified using methyltransferase specific primers G3MetF and G3MetR (Supplementary Table S1) as forward and reverse primers. The expected size of 969 bp in the amplified gene was observed while no amplification was seen in the control (Figure 4A). Further, methyltransferase protein was detected through Western blotting probed with epitope-specific antibody (Genscript). A band of 35 kDa appeared (Figure 4B), which matched the size of the earlier expressed methyltransferase using baculovirus expression system (Sehgal et al., 2006), and also seen in HepG2 cells (Panda et al., 2000). To validate its presence, Immunofluorescence was performed on the uninfected, and the infected cells using primary antibodies of MeT and challenged with goat Anti-Rabbit antibodies conjugated with Alexa flour 488 (Figure 4C). Panels a and d represent uninfected and infected cells stained using DAPI, Panels b and e represent fluorescence of uninfected and infected cells stained with methyltransferase specific antibodies while panels c and f are merged representation of a, b and d, e, respectively. As compared to the control, the fluorescence of the infected cells increased significantly as quantified by using ImageJ (Figure 4D). After confirming the presence of methyltransferase in the infected cell lysate, using immunological characterization, the enzyme was probed for its activity to authenticate its functional role in guanosine methylation leading to the formation of the cap. To check if methyltransferase is functional in the cells, an enzyme assay was performed using MTase-Glo reagent (Promega), mentioned in Section “Materials and Methods.” It was found that methyltransferase activity increased with an increase in the enzyme concentration as compared to mock (uninfected cell) lysate (Figure 4E). To our understanding, this is the first report showing methyltransferase activity in the mammalian cells infected with HEV.

#### Cysteine Protease

**FIGURE 5:**
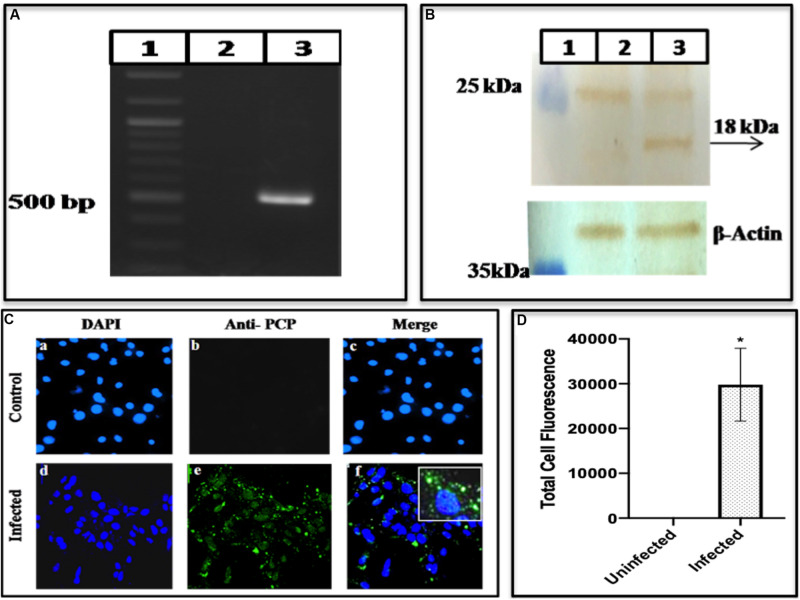
Expression of HEV cysteine protease. **(A)** It represents PCR amplification of Cysteine Protease from BacMam-HEV infected cells using primers G3CPF and G3CPR (Supplementary Table S1). The corresponding lanes are as follows: Marker (lane 1), cDNA from uninfected cells (lane 2), cDNA from infected cells (lane 3). **(B)** Western blotting analysis using Anti-Cysteine Protease antibody and Goat Anti-Rabbit IgG HRP conjugated secondary antibody. The corresponding lanes are as follows: Marker (lane 1), cell lysate from Huh7 control cells (lane 2), cell lysate from infected Huh7 cells showing specific band identical to the size of HEV Cysteine Protease (lane 3). **(C)** Immunofluorescence assay: Cells were stained with Anti-Cysteine Protease primary antibody, which was detected with Alexa Fluor 488 Goat Anti-Rabbit IgG secondary antibody and counterstained with DAPI to stain nuclei. Panels **(a–c)** represent control Huh7 cells and panels **(d–f)** represent HEV infected cells. Panels **(c,f)** represent the merged image. **(D)** The graph represents the mean fluorescence intensity, and the error bar indicates the standard deviation. The statistical significance was tested by the Mann–Whitney test (*p*-value = 0.0286). * indicates *p*-value < 0.05. All the experiments were performed at least three times as independent experiments.

Having established the presence of methyltransferase protein, cleaved from the polyprotein, the presence of the HEV Cysteine Protease was seen which supposedly digests the polyprotein to release its products. PCP gene in the cells was confirmed by amplifying the gene using PCP specific primers G3CPF and G3CPR as forward and reverse primer, respectively (Supplementary Table S1). A band of the required length of ∼483 bases was seen on the gel (Figure 5A lane 3) confirming the presence of PCP gene produced during HEV replication in the cells. The infected cell lysate was fractionated on SDS-PAGE, followed by Western blotting. When the blot was probed with epitope-specific PCP antibodies, an apparent band of 18 kDa was seen (Figure 5B lane 3). The protein was also visualized using Immunofluorescence. While the uninfected cells did not show any fluorescence on antibody staining (upper panel of Figure 5C), an apparent fluorescence on the antibody staining was seen in case of the infected cells (lower panel of Figure 5C) which was quantified and found to be greater in case of infected cells as compared to the uninfected cells (Figure 5D).

#### RNA-Dependent RNA Polymerase

**FIGURE 6:**
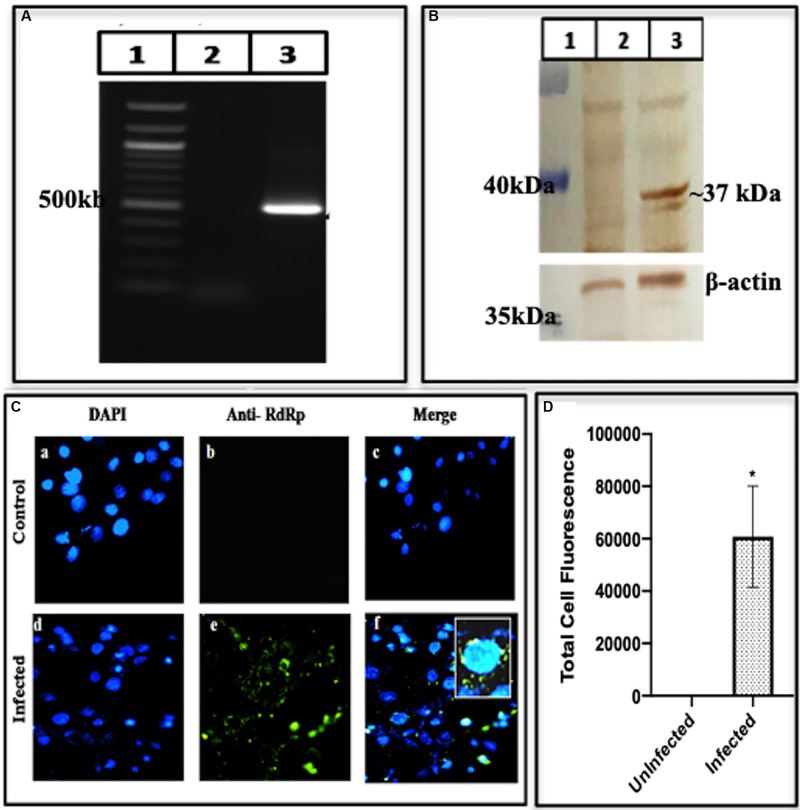
Expression of RNA-dependent RNA polymerase. **(A)** PCR amplification of RdRp from BacMam-HEV infected cells using RdRp specific primers (Supplementary Table S1). The corresponding lanes are as follows: marker (lane 1), cDNA from uninfected cells (lane 2), cDNA from infected cells (lane 3). **(B)** Western blot analysis using anti-RdRp Antibody and Goat Anti-Rabbit IgG HRP conjugated secondary antibody. The corresponding lanes are as follows: marker (lane 1), cell lysate from Huh7 control cells (lane 2), cell lysate from infected Huh7 cells showing specific band identical to the size of HEV RNA dependent RNA polymerase (lane 3). **(C)** Immunofluorescence assay: cells were stained with Anti-RdRp Primary Antibody detected with Alexa Fluor 488 Goat Anti-Rabbit IgG secondary antibody and counterstained with DAPI to stain nuclei. Panels **(a–c)** represent control Huh cells and panels **(d–f)** represent HEV infected cells. Panels **(c,f)** represent the merged image of **(a,b)** and **(d,f)**, respectively. **(D)** The graph represents the mean fluorescence intensity, and the error bar indicates the standard deviation. The data was significantly tested using Mann–Whitney test (*p*-value = 0.0286). * indicates *p*-value < 0.05. All experiments were performed at least three times as independent experiments.

To authenticate the HEV replication, we also looked for the presence of the enzyme polymerase, (RdRp) required for multiplication of the RNA copies. We conducted the RdRp gene amplification and identified the amplified product to be 480 bp (Figure 6A lane 3) while lanes 1 and 2 represented the marker and the mock having uninfected cells. To confirm the presence of RdRp enzyme, Western blotting of the cell lysate was performed using RdRp specific antibodies. Presence of the RdRp of size ∼37 kDa was seen in Figure 6B lane 3, while lanes 1 and 2 show the marker and the mock, respectively. To confirm the presence of RdRp inside the cells, we conducted Immunofluorescence. While the uninfected cells did not show any fluorescence on antibody staining (upper panel of Figure 6C), an apparent fluorescence on the antibody staining was seen in the infected cells (lower panel of Figure 6C).

#### HEV-ORF2 Capsid Protein

**FIGURE 7:**
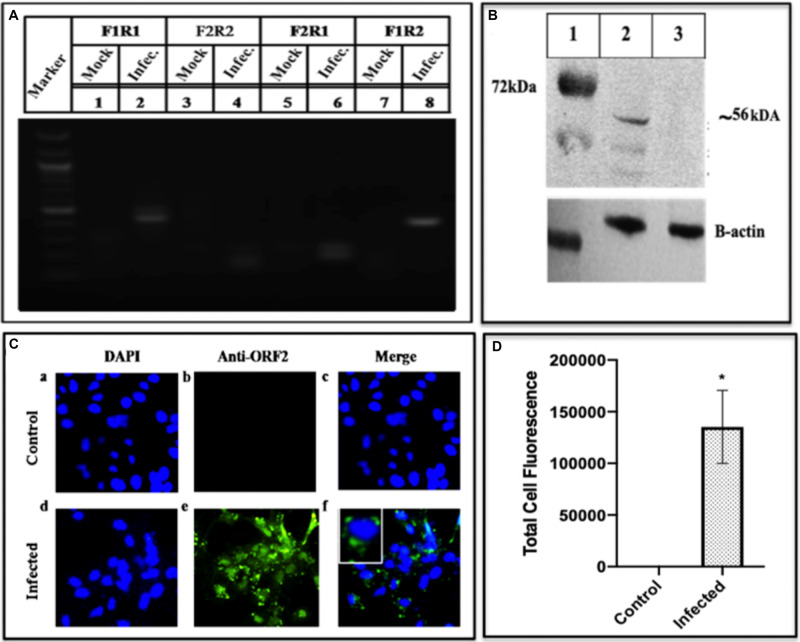
Detection of HEV pORF2 using PCR, western blotting and immunofluorescence. **(A)** The HEV ORF2 gene was amplified from the cell lysate using a different set of ORF2 primers. The corresponding lanes are as follows: marker (lane M), lanes 1, 3, 5, and 7 are the negative controls while lanes 2, 4, 6, and 8 are the fragments amplified with primers from different regions of ORF2 gene as mentioned in Supplementary Table S1. **(B)** Western blot of the cell lysate using epitope-specific ORF2 antibodies. The corresponding lanes are as follows: marker (lane 1), ORF2 protein from infected cells (lane 2), mock (protein from uninfected cells) (lane 3). **(C)** Detection of HEV pORF2 using Immunofluorescence. Cells were stained with Anti-ORF2 primary antibody detected with Alexa Fluor 488 Goat Anti-Rabbit IgG secondary antibody and counterstained with DAPI to stain nuclei. Panels **(a–c)** represent uninfected Huh7 cells and panels **(d–f)** represent HEV infected cells. Panels **(c,f)** represent the merged image of **(a,b)** and **(d,f)**, respectively. **(D)** The graph represents the mean fluorescence intensity, and the error bar indicates the standard deviation. Statistical significance of data was tested using the Mann–Whitney test (*p*-value = 0.0286). * indicates *p*-value < 0.05. All experiments were performed at least three times as independent experiments.

ORF2 forms the capsid of HEV and hence it is the obligate marker of virus multiplication. Presence of the ORF2 transcripts was identified by amplifying the cDNA from infected cells and using the different set of primers specific to different ORF2 regions. F1R1 region amplified using GT3F and GT3R primer, F2R2 amplified using GT3qRTF and GT3qRTR primer, F2R1 amplified using GT3qRTF and GT3R and F1R2 was amplified using GT3F and GT3qRTR forward and reverse primer, respectively, as mentioned in Supplementary Table S1. The sharp amplified products corresponding to the areas between the forward and reverse primers were seen on the gel (Figure 7A lanes 2, 4, 6, and 8). The presence of the transcripts understandably led to translated products, which was confirmed by fractionating the infected cell lysate on the SDS PAGE followed by Western blot that was probed with epitope-specific antibodies of ORF2. Three forms of ORF2 56, 53, and 46 kDa were seen (Figure 7B). The presence of ORF2 protein in the Huh7 cells was further confirmed by using Immunofluorescence by using epitope-specific antibodies against ORF2 (obtained from Genscript) and challenging with secondary antibodies labeled with Alexa Fluor 488. When seen under a fluorescent microscope, the composite image showed the nucleus with DAPI color while those with ORF2 showed green color (Figure 7C). While the uninfected cells did not show any fluorescence on antibody staining (upper panel of Figure 7C), an apparent fluorescence was seen in the infected cells (lower panel of Figure 7C). The results confirmed the presence of ORF2 as the translation product in the virally infected cells.

## Discussion

The puzzle of polyprotein processing remains unsolved due to the absence of an efficient culture system, despite many studies conducted using different conditions by using different expression systems or the viral strains and varying other parameters. Many cell lines like A549, HepG2, PLC/PRF5, Huh7 from various sources like lung, kidney or fibroblasts have been used and found permissive for viral propagation. An approach using different strains of HEV GT 1, 3, or 4 have been reported to be permissive when PLC/PRF/5 and A549 cell lines were used to replicate the virus (Tanaka et al., 2007). Though the level of replication could be increased, still different expression systems like bacteria, yeast, cell-free, and mammalian have been attempted which resulted in the appearance of the replicative intermediates during the replication but a system to study polyprotein processing needs to be evolved. Hence, we used a novel BacMam approach, to affect the smooth entry of the HEV genome into Huh7 cells. This ensured safe entry of HEV genome inside the cells avoiding the chemical-mediated transfection or electroporation which may be injurious. Secondly, we used epitope-specific (∼10 amino acids), antibodies against all the proteins and enzymes (Supplementary Table S2). The transduction of the viral genome into Huh7 cells was performed through BacMam strategy in which the genome was cloned in pDEST-BacMam vector (Figure 1) under the CMV promoter. Consequently, the recombinant BacMam carrying HEV genome was used to infect Huh7 cells to form the HEV and effect its replication (see section “Materials and Methods”). A time-course study was performed to study the appearance of two species of (+) and (−) RNA as a function of time. Around 8.3 × 10^3^ RNA copies of RNA were seen at 8 h post-infection which increased to 5.2 × 10^5^ at 48 h (Figure 2A). During the similar study, when GT3 isolate from a hepatitis patient was transfected in PLC/PRF/5, and A549 cells, 10^7^ copies/ml were produced, which peaked at 50 days post-transfection. In another cell culture system, HEV RNA peaked at sixth-passage showing 1.5 × 10^8^ copies/ml on 10 days post-infection (Tanaka et al., 2007; Yamada et al., 2009b). Though the titre in our current study was low and peaked at 48 h, this might have avoided the degradation of translated proteins which could be seen.

Our system could generate (+) and (−) stranded RNA besides the hybrid RNA, an intermediate of replication (Figures 2D,E, respectively). These RNA species have also been detected earlier in hepatic cells by using strand-specific RT-PCR of HEV RNA (Varma et al., 2011). Similar RNA species were also detected in pigs when infected with a swine strain of HEV (Williams et al., 2001). With the same species of RNA being present, the BacMam HEV culture system was found to be cross- infectious since the cell lysate could infect the healthy Huh7 cells (Figure 3). Earlier, in a successful experiment on processing, the transfection of HepG2 cells by HEV cDNA resulted in fragments of 35, 38, and 36 kDa representing Met, Hel, and RdRp domains, respectively (Panda et al., 2000). Partially matching, we could also detect the fragments of sizes 18, 35, 37, and 56 kDa representing PCP, MeT, RdRp and ORF2, respectively (Figures 4–7). The exact size of 35 kDa was found for methyltransferase (Figure 4B) in our study as well. The same-sized fragment has been shown in an earlier study using the Baculovirus Expression Vector System. The molecular weight was confirmed using MALDI-TOF, and the protein was identified using anti-His antibodies (Sehgal et al., 2006) and confirmed through their current studies (Saraswat et al., 2020). The same size of the enzyme has been earlier predicted through computational analysis by Koonin et al., 1992. Since methyltransferase is the enzyme that methylates the guanine, we checked its activity in the infected cell lysate using a commercial kit (Promega). The enzyme was found to be active, and its activity increased with an increase in the enzyme concentration (Figure 4E). Earlier, the guanine-7-methyltransferase activity of enzyme expressed in the Baculovirus expression system has been attributed to a 110 kDa protein (P110) (Magden et al., 2001). The P110 kDa protein fragment overlaps the region of 35 kDa, and hence the activity could be attributed to the part which is possibly the domain conferring activity on the 110 kDa region. Authenticity about the enzymatic nature of methyltransferase could be confirmed since the increase in its amount resulted a rise in the activity (Figure 4E).

A bottleneck in understanding the processing of polyprotein is due to a lack of our knowledge about the role of Cysteine Protease and its cleavage into smaller proteins. In a recent study, purified PCP was expressed in insect cells and tested for digestion of the bacterially expressed HEV-ORF1. The protease activity was seen using standard assays and further confirmed through site-specific inhibitors, identified through computational modeling and MD simulations (Saraswat et al., 2020). The similar-sized Protease has been seen in the BacMam infected Huh7 cells (Figure 5B). Another key enzyme that is required for HEV replication is the RNA dependent RNA polymerase has been detected as a translational product in our study by using enzyme specific antibodies. Using Western blotting and Immunofluorescence, we could identify this fragment having a molecular weight of ∼37 kDa (Figure 6B) though its activity could not be confirmed due to its low expression. RdRp has been reported to be of varied size in different systems, e.g., it is seen as 63 kDa in the *E. coli* system (Mahilkar et al., 2016) and 48 kDa (Agrawal et al., 2001) while 37 kDa in HepG2 cells (Panda et al., 2000) respectively. Besides the non-structural polyprotein processing, we also checked the same for HEV-capsid ORF2 protein. The infected cell lysate represented the fragments of size ∼56, ∼53, and ∼46 kDa as the processed forms, determined using Western blotting (Figure 7B) (Robinson et al., 1998; Sehgal et al., 2003).

In future, a more efficient culture system for HEV will lead to the progress in our understanding of the mechanism of polyprotein processing. A challenge would be to express the active Cysteine Protease along with the determination of its structural analysis. Determination of the activity of all the viral enzymes will help in testing the drugs or the inhibitors against the HEV. In the present study, we could show that HEV replication has been affected by the presence of Ribavirin as a function of time. Clinical relevance of the Ribavirin is debatable though the drug has been suggested to be a potential therapy against HEV as the off-label drug. A retrospective study has reported that acute hepatitis due to HEV genotype 3 could be treated with Ribavirin (Kamar et al., 2010; Goyal et al., 2012) though it is not true in the case of pregnancy (Gupta and Lama, 2017). Besides, Ribavirin could be dangerous because of mutations in the hypervariable region and ORF1 of HEV that have been reported (Todt et al., 2016, 2018). We conclude that a more efficient HEV culture system can further substantiate the cleavage of HEV proteins and find their action and inhibition through different drugs.

## Materials and Methods

### Cell Culture

The Spodoptera frugiperda (Sf21) cell line (Invitrogen) was maintained at 27°C in the refrigerated incubator for the generation of Recombinant BacMam. The cells were grown in Gibco^TM^ Sf-900^TM^ III Serum-free medium supplemented with heat-inactivated 10% Fetal Bovine Serum (FBS, Qualified, Brazil Origin, Gibco). The Huh7 cell line (a kind gift from Shahid Jameel) was maintained at 37°C in a humidified incubator at 5% CO_2_. These cells were grown in DMEM (Dulbecco’s Modified Eagle Medium, Gibco), which had 10% heat-inactivated FBS and 1× PenStrep (Invitrogen).

### Construction of Recombinant BacMam-HEV

Full-length genome of 7.2 Kb HEV cDNA was amplified using Platinum PCR SuperMix High Fidelity (Invitrogen) from the cDNA clone of swine HEV (pSHEV-3, Kind gift from Meng, Accession No. AY575859). The PCR fragment of the HEV genome was cloned in the intermediate pDONR 221 vector (Invitrogen) to make entry clone which was confirmed by PCR, restriction digestion and, partial sequencing. The HEV genome from entry clone was transferred to the destination vector, BacMam-pCMV-DEST vector (Invitrogen) using Gateway cloning by manipulating the entry and donor vector (Hartley et al., 2000). The recombinant vector, BacMam-pCMV-DEST-HEV, was confirmed for the presence of HEV by PCR using HEV specific primer and transformed into the Gibco^TM^ MAX Efficiency^TM^ DH10Bac Competent Cells which contains a baculovirus shuttle vector called bacmid. Transposition occurred between the Tn elements of the recombinant BacMam vector and the Bacmid DNA leading to the integration of the HEV genome, along with CMV promoter, into the bacmid genome. The DNA from recombinant bacmid was supernatanted (PureLink^TM^ HiPure Plasmid Filter Maxiprep Kit, Invitrogen) and transfected into Sf21 cells using cellfectin (Invitrogen) and kept at 27°C for 96 h to generate recombinant baculovirus carrying HEV genome under CMV promoter. The 1 ml medium was used to infect a T-175 flask containing Sf21 cells in 25 ml of Sf-900 III medium (Invitrogen) under the conditions mentioned above. After 96 h when all signs of infection appeared the recombinant baculovirus, having HEV genome under CMV promoter (BacMam-HEV) was harvested and titered using plaque assay and stored in aliquots at −20°C or −70°C. BacMam-HEV was used for transduction of Huh7 cells at MOI of ∼100. Briefly, the 3 × 10^5^ Huh7 cells were seeded in six-well plate that was incubated at 37°C for 24 h under 5% CO_2_. Baculoviral particles 3 × 10^7^were overlaid on the cells at MOI of 100 and allowed to adsorb for 4 h. After incubation, media was replaced with complete DMEM medium containing 10% FBS and left at 37°C in a CO_2_ incubator for 48 h.

### Detection of HEV Replication

Signs of replication were studied by detecting positive RNA, negative RNA, and the hybrid RNA of the positive and negative strand. To identify the HEV RNA in recombinant baculovirus transduced Huh7 cells, strand-specific RT-PCR was performed using ORF2 primers (Supplementary Table S1). Huh7 cells were grown in one of the wells of a six-well plate and resuspended in 400 μl trizol reagent (Invitrogen) to incubate for 5 min at RT. After incubation, RNA was precipitated using isopropanol and pelleted by centrifuging at 12,000 rpm for 15 min at 4°C. The pelleted RNA was dissolved and incubated with DNase I enzyme (Invitrogen) for 30 min at 37°C to make RNA free from any DNA contamination. The DNase enzyme was inactivated by adding 1 μl of EDTA (0.5 M) and incubating at 65°C for 10 min. The DNase free RNA was used as the template for cDNA synthesized by HEV specific primer of the ORF2 region according to the protocol mentioned in verso cDNA synthesis kit (Invitrogen). The resulting cDNA was used for PCR amplification by the forward and reverse primers of the ORF2 region to detect negative and positive strands of the RNA. The PCR was performed for 35 cycles of denaturation (95°C; 30 s), annealing (56°C; 30 s), and extension (72°C; 30 s) and the product was analyzed on 1.4% agarose gel. Real-time PCR was conducted using SYBR green-based dye (Invitrogen) and detected by Bio-Rad System (Bio-Rad, Hercules, CA, United States) using standard PCR conditions. The method for quantification of HEV copy number was adapted from Debing et al. (2014). The concentration of the amplified product of the HEV genome was measured using nanodrop to calculate the number of copies of the amplified HEV product (copies/μl). Aliquots of DNA were prepared in 10-fold serial dilution from 1 × 10^8^ to 10 copies per μl and, 1 μl of standard dilution of each stock was used for the qPCR reaction. The observed *C*q value for each standard was plotted on *Y*-axis and copies/μl was plotted on *X*-axis. The standard curve was determined using the equation *Y* = −3.176x + 37.58. Viral RNA quantification was performed in three different experiments and in triplicates. After detecting positive and negative-stranded RNAs using forward and reverse primers of ORF2, the hybrid RNA was detected by J2 monoclonal antibody (Biogenuix) which picks up the double-stranded RNA only (Weber et al., 2006) by Immunofluorescence (IF). The detailed protocol of Immunofluorescence has been mentioned in further section.

### Cross-Infectivity Assay

To confirm the *in vitro* virus replication, the cross-infectivity assay was performed. Briefly, infected cells from T-25 flask were harvested and freeze-thaw three times by incubating at −80°C and room temperature as described earlier (Parvez et al., 2011; Córdoba et al., 2012). The resulting lysate was used to infect the uninfected Huh7 cells by incubating with the virus for 48 h at 37° in a CO_2_ incubator. The cross-infection was determined by measuring the HEV copy number using qPCR by GT3qRTF and GT3qRTR primer (Supplementary Table S1) from the lysate of cross-infected Huh7 cells. Huh7 cells were treated with Ribavirin using following protocol.

Huh7 cells (0.3 × 10^6^) were seeded in each well of a six-well plate and incubated at 37°C for 24 h under 5% CO_2_ after which they were infected with the cell lysate of transduced cells (as mentioned above). One ml of complete DMEM medium was added to the cells in two wells, one with and other without 100 μM Ribavirin and incubated. After 24 and 48 h of infection, the cells were harvested and the RNA copy number was calculated in both using qPCR. The experiment was performed at least three times and in duplicates. Further, the cross-infectivity was authenticated using Immunofluorescence (mentioned in Immunofluorescence section).

### Western Blot Analysis

Approximately, 1 × 10^6^ Huh7 cells transduced with recombinant BacMam-HEV were kept for 48 h at 37°C after which they were harvested using 0.25% trypsin (Invitrogen) followed by neutralization with complete medium. The cells were centrifuged at 1,500 rpm for 10 min, and the medium was discarded. The Western blot was performed as described earlier (Devhare et al., 2013) with minor modification in the protocol. Briefly, the cells were washed with 400 μl PBS and suspended in 100 μl of RIPA buffer (Sigma), containing 150 mM NaCl, 50 mM Tris pH 8.0, 1% NP-40, 0.5% deoxycholate and, 0.1% SDS including Protease inhibitors cocktail (Ameresco). The 4 μg of proteins from the cell lysate was resolved on 10% SDS-PAGE and transferred onto PVDF membranes (Millipore). The membrane was blocked with PBS containing 3% BSA, and 0.1% Tween-20 for 1 h at room temperature. After blocking, the membrane was incubated individually with the epitope-specific anti-ORF2, anti-Met, anti-PCP and anti-RdRp rabbit antibody as the primary antibody (Genscript, United States) at a 1:1500 dilution for overnight at 4°C (Supplementary Table S2). Subsequently, the membrane was washed with 5 ml of PBST and incubated with 1:3000 dilutions of HRP-conjugated goat Anti-Rabbit IgG (Invitrogen) and developed with DAB (Sigma) or with Clarity, Max ECL Western blotting substrate (Bio-Rad).

### Immunofluorescence Assay

Translation products of the HEV were confirmed by Immunofluorescence to detect the presence of HEV enzymes and proteins in infected cells. 3 × 10^5^ Huh7 cells were grown on coverslips and infected with HEV expressing recombinant baculovirus at an MOI of 100 and incubated at 37°C for 48 h in a CO_2_ incubator. The transduced cells were washed in PBST and fixed for 10 min in 4% paraformaldehyde. at RT with After fixation, the cells were washed twice with PBS and made permeable for10 min with 0.5% Triton X-100 in PBS at RT. The cells were washed with PBST and blocked with 3% BSA followed by a 1-h incubation with primary antibodies at a dilution of 1:1000 at RT. The same protocol was followed for IF of ORF2, methyltransferase, RdRp, PCP and double-stranded RNA by using antibodies of 1:1000 dilutions. The cells were incubated for 2 h at room temperature followed by 2°Ab (Alexa Flour 488 goat anti-mouse Abcam 1:1000 for ds-RNA detection and Alexa flour 488 Anti-Rabbit 1:1000 for HEV protein detection) for 2 h. The cells were stained in mounting media containing DAPI (Invitrogen), and images were captured using a Leica Fluorescent Microscope. All the Immunofluorescence experiments were performed at least three times as independent experiments.

### Methyltransferase Assay

The assay was performed using MTase-Glo kit (Promega). Methyltransferase converts SAM to SAH (*S*-adenosyl homocysteine) which is converted to ADP by the MTase-Glo^TM^ Reagent. This ADP is converted to ATP by MTase-Glo^TM^ detection Solution. ATP generated in this process is detected through luminescence. To determine methyltransferase activity, 1,000 ng of RNA was incubated with 10 μM SAM (*S*-adenosyl-L-methionine) and at a concentration 0.3–4.8 μg of cell lysate of infected Huh7 cells for 2 h at 37°C. A similar reaction was performed with the lysate of uninfected Huh7 cells as a negative control. After completion of methylation reaction, the 5 μl of MTase-Glo^TM^ Reagent was added to convert SAH to ADP and incubated for 30 min and thee luminescence was measured by a luminometer. The methyltransferase activity was performed at least three times as different experiments and in triplicates.

### Quantification of Fluorescence

In order to quantify the fluorescence of Immunofluorescent cells, an ImageJ software was used^1^. Experimentally, the level of total cell fluorescence was estimated by selecting the cell of interest using the circle as a selection tool. To quantify the fluorescence intensity, four most fluorescing cells were selected in each experiment. The area, integrated density and mean gray value were calculated, and the values were substituted in the following equation: CTCF = Integrated density − (Area of selected cell × Mean fluorescent of background readings) (McCloy et al., 2014). This led to the quantification of the total cell fluorescence, which was plotted against the infected and uninfected cells. To note that since the fluorescence was calculated in four different cells, it had a marginally higher standard deviation.

### Statistical Analysis

Numerical data were tested for statistical significance using 2 way ANOVA and the Immunofluorescence data was tested using the Mann–Whitney test. The analysis is provided in the Supplementary Material. All data were analyzed by Graph pad-Prism (version 8.3.0).

## Data Availability Statement

The datasets generated for this study are available on request to the corresponding author.

## Author Contributions

DS contributed to conceptualization, designing, supervision, finding acquisition, and manuscript preparation. ManK and PH contributed to designing, conducting the experiments, analysis, and drafting the manuscript. MadK acquired the funding. UP worked on the experiment, imaging, and analysis. All authors contributed to the article and approved the submitted version.

## Conflict of Interest

The authors declare that the research was conducted in the absence of any commercial or financial relationships that could be construed as a potential conflict of interest.

## Footnotes

1 1http://rsbweb.nih.gov/ij/download.html
