# Supplementary material for: Development of BacMam Induced Hepatitis E Virus Replication Model in Hepatoma Cells to Study the Polyprotein Processing
Source: Front Microbiol. 2020 Jun 18;11:1347. doi: 10.3389/fmicb.2020.01347 (PMC7315041; doi:10.3389/fmicb.2020.01347)
Supplement: Supplementary file 1 [file Data_Sheet_1.ZIP › Supplementary Files/Tables.docx]

**Supplementary data**

**Table 1:** List of Primers used in this study

| **S.No.** | **Name of Primer** | **Sequence of the primer** |
| --- | --- | --- |
| **1** | GT3pDONR1 | 5' GGGGACAAGTTTGTACAAAAAAGCAGGCTGGCAGACCACGTATGT3' |
| **2.** | GT3pDONR1 | 5'GGGGACCACTTTGTACAAGAAAGCTGGGTTTTTTTTTTTTTTTTCCAGG3' |
| **3.** | GT3RTF | 5' AGCTCCTGTACCTGATGTTGACTCACGTGGT 3' |
| **4.** | GT3RTR | 5' CTACAGAGCGCCAGCCTTGATTGCGGT 3' |
| **5.** | GT3qRTF | 5' GTGGCTATGCTATCTCCATTTCT 3' |
| **6.** | GT3qRTR | 5' GATGACTAACTCGGAGGCAATAC 3' |
| **7** | G3MetF | 5' GTGGTTCGGCCGTTTTTATCTCGTG 3' |
| **8** | G3MetR | 5' CGCACCGACAGTGACCTTGTAGCTAAT 3' |
| **9** | G3CPF | 5' GCACAGTGCCGGCGGTGGTTAT 3' |
| **10** | G3CPR | 5' TAGGACATACTGCTCAGGACCATTCGC 3' |
| **11** | G3RPF | 5' AAGGTTGGTCAGGGTATATCGGCCTG 3' |
| **12** | G3RPR | 5' ACAAAGGACCACCGAATCATCACCCT 3' |

**Table 2**: Peptide sequences used to raise antibodies against HEV proteins mentioned above. (Saraswat et al., 2020)

| **Serial No.** | **Protein** | **Amino Acid Sequence** |
| --- | --- | --- |
| **1** | PCP | LDPRVLVFDESAPC |
| **2** | MeT | AGRDVQRWYTAPTRC |
| **3** | ORF 2 | QQDKGIAIPHDIDLC |
| **4** | RdRp | PKESLKGFWKKHSG |

Saraswat, S., Chaudhary, M., and Sehgal, D. (2020). Hepatitis E Virus Cysteine Protease Has Papain Like Properties Validated by in silico Modeling and Cell-Free Inhibition Assays. 9, 1–16. doi:10.3389/fcimb.2019.00478.
