# Supplementary material for: Development of BacMam Induced Hepatitis E Virus Replication Model in Hepatoma Cells to Study the Polyprotein Processing
Source: Front Microbiol. 2020 Jun 18;11:1347. doi: 10.3389/fmicb.2020.01347 (PMC7315041; doi:10.3389/fmicb.2020.01347)
Supplement: Supplementary file 1 [file Data_Sheet_1.ZIP › Supplementary Files/Antibody CoA/RdRP-COA.pdf]

GenScript

Make Research Easy

CONFIDENTIAL

Certificate of Analysis

CONFIDENTIAL

SC1649 PolyExpress Gold Package

Order ID: U2769DE110-12

Report From:

GenScript USA Inc

860 Centennial Ave

Piscataway, NJ 08854

United States of America

For research use only

CONFIDENTIAL

GenScript

Make Research Easy
CONFIDENTIAL

1. Project Information

Order ID: U2769DE110-12
Antigen Name: RdRP
Sequence: CPKESLKGFWKKHSG (Lot: U9489DE110-1)
Immunogen: Peptide-KLH conjugate
Host Strain: New Zealand Rabbit

2. Product Information(s)

	Detail	Form	Concentration (mg/ml)	Volume (ml)	Quantity (mg)	Purity	Aliquot	Preservative	Buffer
**Product 1**	Antigen (U9489DE110-1)	Lyophilized	/	/	2.00	/	1	/	/
**Product 2**	Pre-immune serum	Lyophilized	/	0.50	/	/	1	0.02% Sodium Azide	/
**Product 3**	Affinity-Purified antibody	Lyophilized	0.484	6.00	2.90	≥98 %	1	0.02% Sodium Azide	PBS

Buffer PBS is short for Phosphate Buffered Saline (PBS, pH 7.4)
Concentration is measured by NanoDrop Spectrophotometer A280nm
Purity is measured by SDS-PAGE

CONFIDENTIAL

GenScript

Make Research Easy
CONFIDENTIAL

3. QC Results:

Indirect ELISA:

Coating Antigen(s): Free Peptide

Coating Concentration: 4 µg/ml, 100 µl/well

Coating Buffer: Phosphate Buffered Saline, pH7.4

Secondary Antibody: Anti-Rabbit IgG Fc Monoclonal Secondary Antibody (Min X Hu, Ms, Rt, Sh, Bv, Gt, Camel) (HRP conjugate) (GenScript, Cat. No. A01856)

Table 1. ELISA results of pre-immune serum and purified antibody

Concentration (ng/ml)	NC	1,000.00	500.00	250.00	125.00	62.50	31.25	15.62	7.81	3.90	1.95	Blank	/
Dilution	1:1,000	1:1,000	1:2,000	1:4,000	1:8,000	1:16,000	1:32,000	1:64,000	1:128,000	1:256,000	1:512,000	Blank	Titer
Antibody	0.052	2.880	2.793	2.719	2.661	2.543	2.266	1.984	1.461	0.904	0.555	0.070	>1:512,000

The titer is the highest dilution with S/B (Signal/Blank) >=2.1, the OD450 in blank is the average of two technical replicates.
The starting concentration of 1 mg/ml and the corresponding dilution ratio is calculated based on the actual concentration.
NC is negative control (Pre-immune serum)

4. Storage Condition

The antiserum/antibody is stable at 2-8°C for up to 1 month. For long term storage, aliquot the antiserum/antibody and store at -20°C or below. Avoid repeated freeze-thaw cycles. Antiserum/Antibody is stable for up to two years.

CONFIDENTIAL

860 Centennial Ave., Piscataway, NJ 08854, USA
Toll-Free: 1-877-436-7274 Tel: 1-732-885-9188 Fax: 1-732-210-0262 Email: order@genscript.com Web: www.genscript.com

CONFIDENTIAL

Date: 6/15/2018

Approved by

Sherry Xu

Sherry Xu

Project Manager, Antibody Department

GenScript USA Inc
