# Supplementary material for: Development of BacMam Induced Hepatitis E Virus Replication Model in Hepatoma Cells to Study the Polyprotein Processing
Source: Front Microbiol. 2020 Jun 18;11:1347. doi: 10.3389/fmicb.2020.01347 (PMC7315041; doi:10.3389/fmicb.2020.01347)
Supplement: Supplementary file 1 [file Data_Sheet_1.ZIP › Supplementary Files/Antibody CoA/Protein Helicase-COA.pdf]

Certificate of Analysis

CONFIDENTIAL

SC1649 PolyExpress Gold Package

Order ID: U2769DE110-18

Report From:

GenScript USA Inc

860 Centennial Ave

Piscataway, NJ 08854

United States of America

For research use only

CONFIDENTIAL

GenScript

Make Research Easy
CONFIDENTIAL

1. Project Information

Order ID: U2769DE110-18
Antigen Name: Protein Helicase
Sequence: CTADARGLIQSSRAH (Lot: U3556DE110-1)
Immunogen: Peptide-KLH conjugate
Host Strain: New Zealand Rabbit

2. Product Information(s)

	Detail	Form	Concentration (mg/ml)	Volume (ml)	Quantity (mg)	Purity	Aliquot	Preservative	Buffer
**Product 1**	Antigen (U3556DE110-1)	Lyophilized	/	/	2.00	/	1	/	/
**Product 2**	Pre-immune serum	Lyophilized	/	0.50	/	/	1	0.02% Sodium Azide	/
**Product 3**	Affinity-Purified antibody	Lyophilized	0.781	3.80	2.97	≥97 %	1	0.02% Sodium Azide	PBS

Buffer PBS is short for Phosphate Buffered Saline (PBS, pH 7.4)
Concentration is measured by NanoDrop Spectrophotometer A280nm
Purity is measured by SDS-PAGE

CONFIDENTIAL

3. QC Results:

Indirect ELISA:

Coating Antigen(s): Free Peptide

Coating Concentration: 4 µg/ml, 100 µl/well

Coating Buffer: Phosphate Buffered Saline, pH7.4

Secondary Antibody: Anti-Rabbit IgG Fc Monoclonal Secondary Antibody (Min X Hu, Ms, Rt, Sh, Bv, Gt, Camel) (HRP conjugate) (GenScript, Cat. No. A01856)

Table 1. ELISA results of pre-immune serum and purified antibody

Concentration (ng/ml)	NC	1,000.00	500.00	250.00	125.00	62.50	31.25	15.62	7.81	3.90	1.95	Blank	/
**Dilution**	**1:1,000**	**1:1,000**	**1:2,000**	**1:4,000**	**1:8,000**	**1:16,000**	**1:32,000**	**1:64,000**	**1:128,000**	**1:256,000**	**1:512,000**	**Blank**	**Titer**
**Antibody**	0.054	2.762	2.695	2.633	2.531	2.495	2.258	1.953	1.527	1.117	0.631	0.063	>1:512,000

The titer is the highest dilution with S/B (Signal/Blank) >=2.1, the OD450 in blank is the average of two technical replicates.
The starting concentration of 1 mg/ml and the corresponding dilution ratio is calculated based on the actual concentration.
NC is negative control (Pre-immune serum)

4. Storage Condition

The antiserum/antibody is stable at 2-8°C for up to 1 month. For long term storage, aliquot the antiserum/antibody and store at -20°C or below. Avoid repeated freeze-thaw cycles. Antiserum/Antibody is stable for up to two years.

CONFIDENTIAL

CONFIDENTIAL

Date: 6/15/2018

Approved by

Sherry Xu

Sherry Xu

Project Manager, Antibody Department

GenScript USA Inc
